# Anodal Transcranial Direct Current Stimulation Promotes Frontal Compensatory Mechanisms in Healthy Elderly Subjects

**DOI:** 10.3389/fnagi.2017.00420

**Published:** 2017-12-18

**Authors:** Jesús Cespón, Claudia Rodella, Paolo M. Rossini, Carlo Miniussi, Maria C. Pellicciari

**Affiliations:** ^1^Cognitive Neuroscience Section, Istituto di Ricovero e Cura a Carattere Scientifico Centro San Giovanni di Dio Fatebenefratelli, Brescia, Italy; ^2^Institute of Neurology, Policlinico A. Gemelli, Catholic University of the Sacred Heart, Rome, Italy; ^3^Center for Mind/Brain Sciences, University of Trento, Rovereto, Italy

**Keywords:** transcranial direct current stimulation, working memory, event-related potentials, P300, aging, compensatory mechanisms

## Abstract

Recent studies have demonstrated that transcranial direct current stimulation (tDCS) is potentially useful to improve working memory. In the present study, young and elderly subjects performed a working memory task (*n*-back task) during an electroencephalogram recording before and after receiving anodal, cathodal, and sham tDCS over the left dorsolateral prefrontal cortex (DLPFC). We investigated modulations of behavioral performance and electrophysiological correlates of working memory processes (frontal and parietal P300 event-related potentials). A strong tendency to modulated working memory performance was observed after the application of tDCS. In detail, young, but not elderly, subjects benefited from additional practice in the absence of real tDCS, as indicated by their more accurate responses after sham tDCS. The cathodal tDCS had no effect in any group of participants. Importantly, anodal tDCS improved accuracy in elderly. Moreover, increased accuracy after anodal tDCS was correlated with a larger frontal P300 amplitude. These findings suggest that, in elderly subjects, improved working memory after anodal tDCS applied over the left DLPFC may be related to the promotion of frontal compensatory mechanisms, which are related to attentional processes.

## Introduction

Cognitive aging is characterized by patterns of cognitive decline that are specific to each cognitive function in terms of onset and progression rate (Salthouse, 2009; Park and Bischof, 2013). The aging of society is leading to an increased prevalence of chronic diseases, including those affecting cognition, such as Alzheimer’s disease (Sosa-Ortiz et al., 2012). Therefore, the scientific community is currently increasing its effort to diversify pharmacological targets (Cummings et al., 2014) and develop non-pharmacological interventions (Bamidis et al., 2014; Hsu et al., 2015) to treat, prevent, or slow down aging mechanisms that lead to the progression of the cognitive decline characteristic of normal and pathological aging.

Executive control functions decline substantially with physiological aging (Grady, 2012). These functions include a set of cognitive processes—such as working memory, cognitive inhibition, cognitive flexibility, and attentional and inhibitory control—that humans use in daily life activities to successfully monitor behaviors and implement goal-directed actions (Chan et al., 2008; Diamond, 2013). Working memory, an extensively studied executive control function, includes a set of cognitive processes that allow humans to encode, store, maintain, and manipulate information for a short time period (Baddeley, 2003). These cognitive processes become less efficient with age (Park et al., 2002; Peich et al., 2013; Kirova et al., 2015), and this age-related decline has been associated with altered patterns of brain activity and connectivity during the working memory tasks (Cook et al., 2007; Daffner et al., 2011; Sander et al., 2012; Pinal et al., 2015).

A promising tool to slow down cognitive decline is transcranial direct current stimulation (tDCS), which is thought to improve a wide range of cognitive functions by promoting brain plasticity mechanisms (Hsu et al., 2015; Dedoncker et al., 2016; Summers et al., 2016). The tDCS technique consists of applying a constant flow of current between two electrodes at a low intensity (1–2 mA) for about 5–20 min. tDCS modulates cortical excitability by modifying the spontaneous neuronal firing rate (Creutzfeldt et al., 1962). Whereas anodal tDCS increases the spontaneous neuronal firing rate, cathodal tDCS reduces it.

Research focusing on working memory processes has usually applied anodal tDCS over the dorsolateral prefrontal cortex (DLPFC) to improve performance, as the DLPFC is thought to play a crucial role in working memory (Levy and Goldman-Rakic, 2000; Tremblay et al., 2014). A seminal study conducted by Fregni et al. (2005) reported that anodal tDCS over the left DLPFC improved working memory performance in healthy young participants, whereas cathodal tDCS over the left DLPFC and anodal tDCS over the primary motor area did not produce any effect. Afterward, several studies replicated the findings about the improved working memory by applying anodal tDCS over the DLPFC in healthy young subjects (Ohn et al., 2008; Andrews et al., 2011; Keeser et al., 2011; Teo et al., 2011; Lally et al., 2013; Richmond et al., 2014; Carvalho et al., 2015; Au et al., 2016; Talsma et al., 2017) and extended these findings to samples of healthy elderly participants (Berryhill and Jones, 2012; Park et al., 2014; Jones et al., 2015). Nonetheless, some studies reported null effects on cognitive improvement after tDCS was applied over the DLPFC (Mylius et al., 2012; Motohashi et al., 2013; De Putter et al., 2015; Sellers et al., 2015).

The inconsistent results outlined in the previous paragraph may be related to methodological and individual differences across the different studies (Horvath et al., 2014; Fertonani and Miniussi, 2017). In general, meta-analyses of tDCS and working memory have demonstrated that offline tDCS applied to the DLPFC has a moderate impact on working memory functioning in healthy populations (Brunoni and Vanderhasselt, 2014; Hill et al., 2016). This finding is consistent with other meta-analytical studies suggesting that offline stimulation improves cognition more than online stimulation in healthy subjects (Hsu et al., 2015; Dedoncker et al., 2016; Hill et al., 2016). Even so, there exists a set of variables that are able to produce diverse tDCS modulations even if homogeneous samples of subjects are used. For instance, tDCS effects may differ according to individuals’ baseline performance (Tseng et al., 2012; Benwell et al., 2015; Hsu et al., 2016) and/or level of practice in a specific task (Dockery et al., 2009). In this regard, one study found that cathodal tDCS improved performance at the initial stages of training in a motor planning task; however, when participants became relatively skilled, anodal tDCS led to additional improvements, whereas cathodal tDCS led to impaired performance (Dockery et al., 2009). These results were attributed to the tDCS effects on the signal/noise ratio of neural populations involved in performing the task, which depends on the ability to execute the task (Miniussi et al., 2013; Fertonani and Miniussi, 2017). Other studies have also demonstrated that anatomical differences in a sample of healthy young participants affected the spread of current and the concomitant behavioral tDCS modulations (Kim et al., 2014). In contrast, it has been suggested that studies using multiple tDCS sessions are able to improve cognition more than tDCS studies using a single session (Horvath et al., 2015; Au et al., 2016). Nonetheless, it is still possible that a single tDCS session causes neural modulations that are not strong enough to result in behavioral effects. In fact, studies have frequently reported neural changes related to aging (Vallesi and Stuss, 2010), cognitive decline (Cespón et al., 2015), or cognitive interventions implemented in elderly participants (Tusch et al., 2016) in the absence of behavioral differences.

Despite the growing interest in investigating the capability of tDCS to improve cognitive functions, the neural correlates that underlie the modulated performance are still poorly understood. Event-related potentials (ERP) represent a suitable tool to investigate the neural correlates of the cognitive processes that are modulated by applying tDCS because the high temporal resolution of ERP is suitable for the high speed of the cognitive processes taking place during the performance of a cognitive-behavioral task.

Electrophysiological studies about working memory have frequently focused on the P300 ERP (Kok, 2001; Watter et al., 2001; Polich, 2007; Daffner et al., 2011). During working memory tasks, the latency of P300 correlates with the speed of context information update (Polich, 2007). The amplitude of parietal P300 is related to the amount of neural activity allocated to the context information update processes, whereas the amplitude of frontal P300 is related to the allocation of attentional resources to an upcoming stimulus (Fabiani and Friedman, 1995; Friedman et al., 2001; Nieuwenhuis et al., 2005; Polich, 2007; Daffner et al., 2011; Wild-Wall et al., 2011; Saliasi et al., 2013; Tusch et al., 2016). Overall, aging is associated with longer P300 latencies and diminished P300 amplitudes (Polich, 1997; for a review, see Rossini et al., 2007). Nonetheless, according to the reported shift from posterior to anterior activity with age, many studies have found diminished parietal P300 amplitude and increased frontal P300 amplitude related to aging (Friedman et al., 1997; Daffner et al., 2011; Saliasi et al., 2013; van Dinteren et al., 2014), which was interpreted as additional allocation of frontal activity to compensate age-related decline in the cognitive processing supported by posterior areas (Friedman et al., 1997). The only study that investigated ERP modulations in young subjects by applying tDCS reported that improved working memory performance in a 2-back task after anodal tDCS was correlated with increased frontal P300 amplitude (Keeser et al., 2011). However, no previous studies have focused on brain activity modulations related to the improved working memory performance in elderly subjects after tDCS.

**FIGURE 1:**
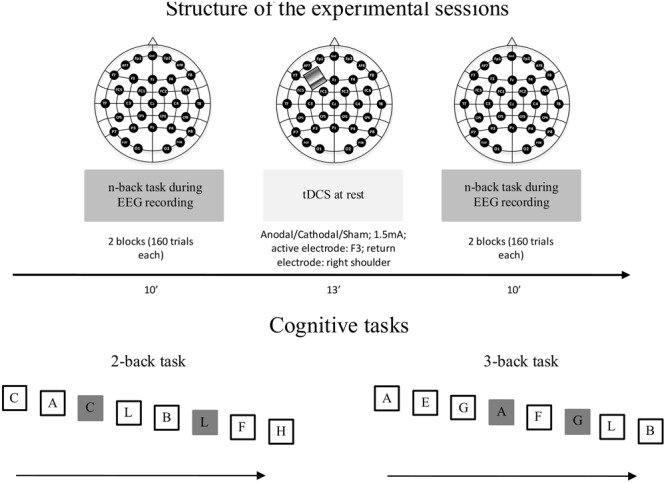
Structure of the experimental sessions (top panel). The participants performed three experimental sessions: sham, cathodal, and anodal tDCS. The sessions were separated by a minimum of 5 days. The order of sessions was counterbalanced between participants. The figure represents the cognitive tasks performed by young (3-back task) and elderly (2-back task) participants (bottom panel). The target letter (25% of trials) is represented within gray squares. Participants responded to the target letter by pressing the space bar. The letters were presented at the center of the screen for 500 ms in white color against a black background. During the inter-stimulus interval (duration jittered at 2000–2500 ms), the screen remained blank.

The aim of the present study was to investigate the capability of tDCS to modulate working memory and underlying neural processes in healthy young and elderly participants, who performed an *n*-back task during an electroencephalogram (EEG) recording before and after anodal, cathodal, and sham tDCS applied over the left DLPFC. To match the task difficulty, young and elderly participants performed a 3-back and a 2-back task, respectively (for a graphic representation of the experimental session and *n*-back tasks, see **Figure 1**).

We hypothesized that the two groups would perform similarly, as elderly subjects performed an easier version of the task. Considering the age-related decline in learning ability (Salthouse, 2009), we expected a greater improvement after sham tDCS in young participants than in elderly participants. In line with most previous studies, we hypothesized that working memory would improve after anodal tDCS in both groups of participants. If this improvement was mediated by the strengthening of attentional mechanisms supported by prefrontal regions, then a larger frontal P300 amplitude would be observed after applying the anodal tDCS. Instead, if this improvement was mediated by more efficient processes related to context information update, then a larger parietal P300 amplitude would be observed after anodal tDCS. Likewise, we were interested in studying whether the possible performance modulations observed after cathodal tDCS were mediated by the modulation of attentional processes and/or processes related to context information update.

## Materials and Methods

### Participants

Fourteen healthy young (six females; mean age = 24.8, SD = 3.69) and 14 healthy elderly participants (nine females; mean age = 70.2, SD = 5.12) took part in the present study. All participants were right-handed, as evaluated using the Edinburgh Handedness Inventory test (Oldfield, 1971). They reported no previous history of neurological or psychiatric disorders and had no metal implants. Furthermore, elderly participants undertook a neuropsychological assessment to ensure that their cognitive functioning was within normal parameters. Experimental protocols were performed in accordance with procedures for non-invasive brain stimulation (Woods et al., 2016). The study was performed in accordance with the ethical guidelines outlined in the 1964 Declaration of Helsinki and received prior approval by The Saint John of God Clinical Research Centre Ethical Committee. The experimental procedures were carefully explained to all participants who volunteered to take part in the study. Informed consent was obtained from all participants. The consent obtained from the participants was both informed and written.

### Procedures

Participants attended three experimental sessions separated by at least 5 days. Participants performed a working memory task (a verbal *n*-back task) before and after tDCS. tDCS was delivered by a battery-driven constant current stimulator (BrainStim, EMS) through two rubber electrodes (anodal area = 16 cm^2^; cathodal area 50 cm^2^). The anode was placed over the scalp overlying the left DLPFC, in correspondence with the F3 electrode and the cathode over the right shoulder. In each experimental session, participants received anodal, cathodal, or sham tDCS. The order of these experimental sessions was counterbalanced across participants. The stimulation ramped up and down for 8 s and remained stable at 1.5 mA for 13 min. In the sham condition, current was delivered for 10 s only at the beginning and at the end of the stimulation block. At the beginning of each experimental session, participants performed a brief practice block. Next, they performed the *n*-back task during the EEG recording (the structure of the experimental sessions is recapped in **Figure 1**).

### Task

The *n*-back task consisted of the presentation of 80 targets and 240 non-targets (i.e., the probability of target appearance was set at 25%) in two separated blocks (40 targets and 120 non-targets per block), each 6 min long. The break between blocks was around 90 s. During the task, the letters A–L randomly appeared in the center of the screen for 500 ms. The letters were presented in white color against a black background. The screen remained blank during the inter-stimuli interval, which was jittered between 2000 and 2500 ms. The screen was placed 100 cm in front of the participants, who were instructed to direct their gaze to the center of the screen throughout the task and to respond, by pressing the space bar, to the stimulus identity if it matched the stimulus that had been presented two trials before (2-back task, which was performed by elderly participants) or three trials before (3-back task, which was performed by young participants). The different versions of the task were created to match the task difficulty level for young and elderly participants. Each participant performed the *n*-back task six times, that is, twice a session (before and after tDCS) in three tDCS sessions (anodal, cathodal, and sham). To prevent participants from learning the letter sequence, the order of stimuli presentation was pseudorandomized so that the letters appeared in a different order each time they performed the task. Before performing the corresponding *n*-back task, participants performed a training block that was 3 min long (20 targets and 60 non-targets). Participants proceeded with the experiment only if they reached 60% accuracy in the practice block, and they could repeat the practice block a maximum of three times.

### EEG Recordings

EEG was recorded using 31 electrodes (Easycap, GmbH, Brain Products) in accordance with the 10–10 International System; these electrodes included Fp1, Fp2, AF7, AF8, F7, F3, Fz, F4, F8, FC5, C1, FC2, FC6, T7, C3, Cz, C4, T8, CP5, CP1, CP2, CP6, P7, P3, Pz, P4, P8, PO7, PO8, O1, and O2. The ground electrode was placed on Fpz. The right mastoid was used as online reference for all electrodes whereas the left mastoid (offline reference) was used to re-reference the activity to the average of the left and right mastoid. The EEG signal was acquired with a 0.1–1000 Hz bandpass filter and digitized at a sampling rate of 5000 Hz (down-sampled to 1000 Hz before ERP pre-processing). Vertical and horizontal eye movements were recorded by two electrodes located above and beneath the right eye and two electrodes located lateral to the external canthi of each eye. Impedance was maintained below 5 kΩs. After signal storage, ocular artifacts were corrected using independent component analysis. The signal was filtered at a 0.1–80 Hz digital bandpass and a 50 Hz notch filter. Epochs exceeding ±100 μV were automatically rejected. All remaining epochs were individually inspected to identify those still displaying artifacts, which were also eliminated from subsequent averaging. Epochs were then corrected to the mean voltage of the 200 ms pre-stimulus recording period (baseline).

### Data Analysis

Performance was evaluated by considering the reaction time (RT) and accuracy. Accuracy was calculated taking into account correct responses and missed responses to the target stimulus as well as erroneous responses to the non-target stimulus (false alarms). This was done using the *d* prime index (*d*′), which was calculated as follows: *d*′ = *Z*_(hit rate)_ -*Z*_(false alarm rate)_, where *Z* represents hit and false alarm rates transformed into *z* scores using the standard normalized probability distribution. A higher *d*′ indicates higher performance. That is, the *d*′ value can be increased by increasing hits to the target stimulus (i.e., accuracy) and/or correct rejections of the non-target stimulus as well as by minimizing the missed responses to the target stimulus or the erroneous responses to the non-target stimulus (i.e., false alarms).

For electrophysiological analyses, ERPs were calculated for the correct responses. The epochs were established between -200 and 800 ms relative to the onset of the target stimulus. P300 ERP was analyzed using the mean amplitude in time windows of 100 ms, ranging from 350 to 550 ms (i.e., 350–450 ms, and 450–550 ms), which was based on the visual inspection of grand averages. Analyses were conducted within four regions of interest (ROIs), which include the stimulated area (i.e., frontal left region), the homologous area (frontal right), and the parietal left and right areas, in which P300 typically achieves maximum amplitudes. The mentioned ROIs were calculated by pooling the following electrodes: frontal left (F3, F7, AF7, FC5), frontal right (F4, F8, AF8, FC6), parietal left (P3, P7, PO7, CP5), and parietal right (P4, P8, PO8, CP6). To understand the functional meaning of the observed ERP modulations, correlation analyses were conducted between P300 changes (i.e., “P300 amplitude after tDCS—P300 amplitude before tDCS”) and *d*′ changes (i.e., “*d*′ after tDCS—*d*′ before tDCS”) for each ROI and experimental condition.

### Statistical Analysis

To evaluate whether tDCS modulated behavioral performance, the corresponding repeated-measures ANOVAs for RTs and *d*′ values were carried out with a between-subject factor, Group (two levels: Young and Elderly) and two within-subject factors, Type of Stimulation (three levels: Anodal, Cathodal, and Sham) and Time (two levels: before tDCS and after tDCS).

For the ERP data, P300 was analyzed using the corresponding repeated-measures ANOVA with a between-subject factor, Group (two levels: Young and Elderly) and two within-subject factors, Stimulation (three levels: Anodal, Cathodal, and Sham) and Time (two levels: before tDCS and after tDCS), for each studied time window (i.e., 350–450 ms, and 450–550 ms) within the corresponding ROIs (i.e., frontal left, frontal right, parietal left, and parietal right). Pearson’s correlation analyses were carried out to analyze the correlation between the magnitude of change in the *d*′ value and the magnitude of change in the P300 amplitude after the different tDCS conditions (i.e., anodal, cathodal, and sham).

The Greenhouse–Geisser correction for degrees of freedom was performed when the condition of sphericity was not met. In these cases, the corresponding degrees of freedom were provided. For significant results, measures of size effect are provided by reporting the partial eta square (η_p_^2^) index. When the ANOVAs revealed significant effects due to the main factors and/or their interactions, *post hoc* comparisons were performed by applying the Bonferroni correction.

## Results

### Behavioral Results

The repeated-measures ANOVA (Group × Stimulation × Time) for RTs revealed a Group effect [*F*(1,26) = 5.08, *p* = 0.033, η_p_^2^ = 0.164], as the RT was faster in young than in elderly participants (*p* = 0.011). The analysis also revealed a Time effect [*F*(1,26) = 7.59, *p* = 0.011, η_p_^2^ = 0.226], as the RTs were faster after tDCS was delivered (*p* = 0.011).

The repeated measures ANOVA (Group × Stimulation × Time) for the *d*′ index revealed a Group effect [*F*(1,26) = 4.39, *p* = 0.046, η_p_^2^ = 0.145], as the *d*′ index was higher in elderly than in young participants (*p* = 0.046). The analysis also revealed a Time effect [*F*(1,26) = 25.2, *p* < 0.001, η_p_^2^ = 0.492], as the *d*′ was higher after tDCS was delivered (*p* < 0.001). In addition, Group × Stimulation × Time revealed a marginally significant effect [*F*(2,52) = 2.83, *p* = 0.068, η_p_^2^ = 0.098]. *Post hoc* comparisons showed that in young participants, *d*′ was higher after sham tDCS (*p* = 0.005) and after cathodal tDCS (*p* = 0.002) but not after anodal tDCS (*p* = 0.420). In contrast, in elderly participants, *d*′ was higher after anodal tDCS (*p* = 0.029) but not after cathodal tDCS (*p* = 0.629) or sham tDCS (*p* = 0.258). Moreover, after anodal tDCS, *d*′ was higher in elderly than in young participants (*p* = 0.042) (*d*′ values are recapped in **Table 1**).

**Table 1:** Means and standard deviations for RT and *d*′ values in young and elderly participants, before (pre) and after (post) tDCS, for all experimental sessions (sham, cathodal, anodal).

		Sham		Cathodal		Anodal	
		Pre	Post	Pre	Post	Pre	Post
Young	RT	735 (218)	698 (215)	722 (185)	689 (174)	753 (234)	712 (213)
	*d*′	1.6 (1.3)	1.9 (1.0)	1.3 (1.6)	1.9 (1.7)	1.5 (1.4)	1.6 (1.5)
Elderly	RT	878 (168)	855 (144)	854 (155)	857 (153)	863 (138)	845 (156)
	*d*′	2.3 (0.7)	2.4 (0.6)	2.2 (0.8)	2.3 (0.7)	2.3 (0.8)	2.6 (0.7)

### ERP Results

**FIGURE 2:**
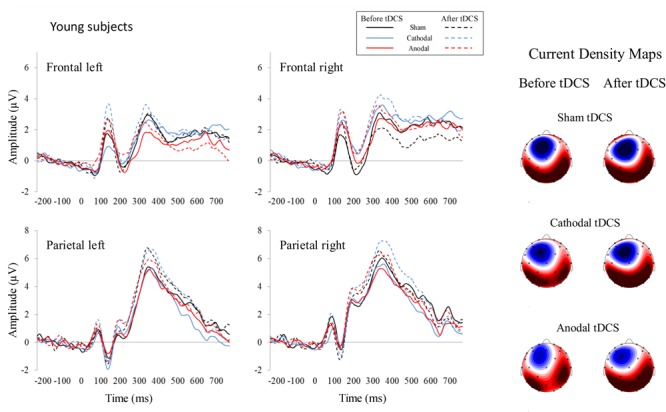
Event-related potentials before and after tDCS in healthy young participants. Each represented waveform results from averaging four electrodes that compounded the respective region of interest: frontal left (F3, F7, AF7, FC5), frontal right (F4, F8, AF8, FC6), parietal left (P3, P7, PO7, CP5), and parietal right (P4, P8, PO8, CP6). Current density maps (350–550 ms) are showed for the three experimental conditions before and after applying the tDCS. These maps revealed a parietal P300 distribution in young subjects.

For the 350–450 ms time window, the repeated-measures ANOVA (Group × Stimulation × Time) within the left frontal region revealed a Time effect [*F*(1,26) = 5.02, *p* = 0.034, η_p_^2^ = 0.162], as the P300 amplitude was larger after than before applying the tDCS (*p* = 0.034). This analysis also revealed a Group × Stimulation × Time interaction effect [*F*(2,52) = 3.94, *p* = 0.026, η_p_^2^ = 0.132]; specifically, in the elderly group, the P300 amplitude was larger after than before anodal tDCS (*p* = 0.001). Moreover, after anodal tDCS, the P300 amplitude was larger in elderly than in young participants (*p* = 0.015). In addition, the P300 amplitude was larger after anodal tDCS than after sham (*p* = 0.019) and cathodal (*p* = 0.003) tDCS. For the right frontal region, the repeated-measures ANOVA (Group × Stimulation × Time) revealed a Group × Stimulation interaction effect [*F*(2,52) = 3.17, *p* = 0.05, η_p_^2^ = 0.132]. Specifically, in elderly, the P300 amplitude was larger after anodal tDCS than after cathodal tDCS (*p* = 0.021). For the left parietal region, the repeated-measures ANOVA (Group × Stimulation × Time) revealed a Group effect [*F*(1,26) = 4.43, *p* = 0.045, η_p_^2^ = 0.146], as the P300 amplitude was larger in young than in elderly (*p* = 0.045). For the right parietal region, the repeated-measures ANOVA (Group × Stimulation × Time) revealed a Time effect [*F*(1,26) = 7.49, *p* = 0.011, η_p_^2^ = 0.224], as the P300 amplitude was larger after than before tDCS (*p* = 0.011). The ERP waveforms are represented in **Figures 2** (young participants) and **3** (elderly participants).

**FIGURE 3:**
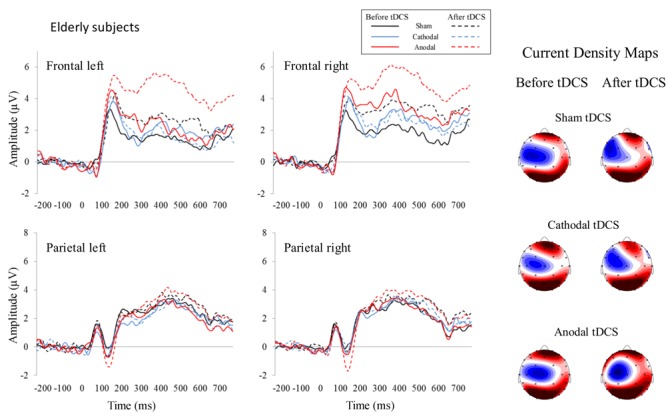
Event-related potentials before and after tDCS in healthy elderly participants. As specified for young subjects, each represented waveform results from averaging four electrodes that compounded the respective region of interest. The amplitude of P300, which is related to working memory processes, was increased in elderly participants after anodal tDCS in the left frontal region at the 350–550 ms time window (see dotted red line). Current density maps (350–550 ms) are showed for the three experimental conditions before and after applying the tDCS. These maps revealed a frontal and parietal P300 distribution in elderly subjects.

For the 450–550 ms time window, the repeated-measures ANOVA (Group × Stimulation × Time) within the left frontal region revealed a significant Group × Stimulation interaction effect [*F*(2,52) = 4.21, *p* = 0.020, η_p_^2^ = 0.139]; specifically, in the anodal tDCS condition, the P300 amplitude was larger in elderly than in young (*p* = 0.027). Also, this analysis revealed a Group × Stimulation × Time interaction effect [*F*(2,52) = 4.61, *p* = 0.014, η_p_^2^ = 0.151]. Specifically, in the elderly group, the P300 amplitude was larger after than before anodal tDCS (*p* = 0.001). Furthermore, in the elderly group, the P300 was larger after anodal tDCS than after sham (*p* = 0.060) and cathodal (*p* = 0.008) tDCS. Moreover, after anodal tDCS, the P300 amplitude was larger in elderly than in young (*p* = 0.003). No significant effects were observed for the right frontal, left parietal, or right parietal regions.

**FIGURE 4:**
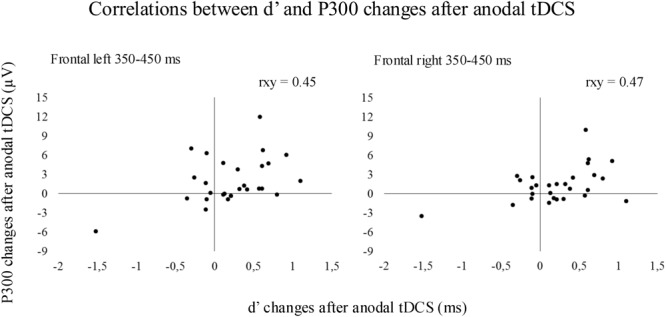
The results revealed significant correlations between the magnitude of the increased *d*′ and the magnitude of the increased P300 at 350–450 ms within frontal regions after anodal tDCS.

Pearson correlation coefficients between enhanced *d*′ values and increased P300 amplitude after tDCS were significant at the 350–450 ms time window within the left and right frontal regions when anodal tDCS was applied (see **Figure 4**). In detail, significant correlations were observed between enhanced *d*′ and increased P300 amplitude after anodal tDCS within the left frontal region (rxy = 0.45, *p* = 0.016) and within the right frontal region (rxy = 0.47, *p* = 0.012). No significant correlations were observed between *d*′ and P300 changes for the 450–550 time window.

## Discussion

**FIGURE 5:**
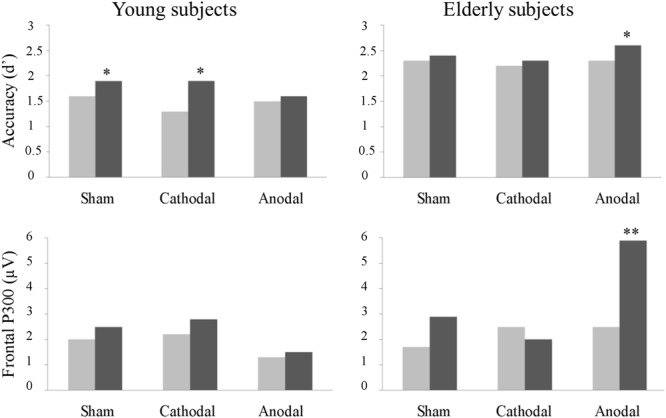
Summary of the main behavioral (top chart) and electrophysiological (bottom chart) results of the present study (^∗^*p* < 0.1, and ^∗∗^*p* < 0.05).

The present study investigated whether and how the anodal and cathodal tDCS delivered over the left DLPFC modulated the performance and the underlying neural activity in young and elderly participants in a working memory task. In the absence of stimulation, young subjects benefited from additional practice in the task, as indicated by improved performance after the sham tDCS. Anodal tDCS induced a working memory improvement in elderly subjects. However, in young, anodal tDCS impeded the spontaneous learning observed in the sham session. No effects were promoted by cathodal tDCS. Anodal tDCS induced a larger frontal P300 component in elderly subjects, which correlated with behavioral (*d*′) improvements. Additionally, the parietal P300 was increased after tDCS, but interactions were not observed between a larger parietal P300 and a specific group or experimental condition. The main results of the study are graphically summarized in **Figure 5**.

Accuracy, measured using the *d*′ index, was higher among elderly than among young, possibly because elderly subjects performed an easier task (2-back task) than did young subjects (3-back task). However, the slower RTs observed in elderly than in young subjects might suggest a trade-off between speed and accuracy among the elderly, which may also explain the greater accuracy observed in this group. Nevertheless, previous ERP studies demonstrated that the age-related slowing in motor execution processes contributes to the slower RTs observed in elderly compared with young subjects even if, as in the present study, speed and accuracy are similarly required of both samples of participants (Kolev et al., 2006; Roggeveen et al., 2007; Cespón et al., 2013).

The behavioral results showed a learning effect related to practice in young but not in elderly subjects, as demonstrated by higher *d*′ after the sham tDCS in the former group. This finding may be related to a greater learning ability of young compared with elderly subjects during the performance of the *n*-back (Salminen et al., 2016) and other cognitive-behavioral tasks (King et al., 2013). Alternatively, these results might suggest the existence of a ceiling effect in elderly subjects, which would prevent a subsequent improvement. However, this possibility should be excluded because an improvement was observed in elderly subjects after the anodal tDCS was applied. In fact, anodal tDCS had opposite effects for young and elderly subjects; anodal tDCS improved the performance of elderly but hindered that of young subjects (who already exhibited improvement without stimulation). As suggested by Bortoletto et al. (2015), it is possible that increased neural excitability related to anodal tDCS disrupted the optimal neural state and impeded the practice-related improvement observed after the sham tDCS. In contrast, cathodal tDCS did not have a behavioral effect in any group. This result suggests that cathodal tDCS did not modulate the neural activity patterns underlying the task performance.

The behavioral results discussed in the previous paragraph deserve additional consideration, as the statistics showed only a tendency (*p* = 0.06) for such differences. These results could be related to the small sample size used in the present study. Moreover, these results probably reflect also the high inter-individual variability in response to the tDCS, as noted by previous studies (Horvath et al., 2014). In fact, a recent meta-analysis reported that offline tDCS applied over the left DLPFC showed no significant but strong tendencies for improved performance in healthy subjects (Hill et al., 2016). Thus, the present results are in line with previous studies. Moreover, these findings warrant further research to identify the individual factors contributing to this variability and encourage investigation about neural correlates of the tDCS modulations.

The main goal of the present study was to investigate the neural processes modulated by tDCS and the neural correlates of the possible behavioral modulations. The electrophysiological results revealed that anodal tDCS increased the left frontal P300 amplitude in elderly participants between 350 and 550 ms. Thus, a larger P300 amplitude can be related to enhanced performance after anodal tDCS, which was also supported by analyses of correlations between the increased P300 amplitude (in the left and right frontal regions between 350 and 450 ms) and the improved *d*′ index after anodal tDCS. These results are consistent with previous investigations that focused on P300 ERP modulations after other types of interventions were applied with the aim to improve cognition. A previous study reported a greater P300 amplitude after 5 weeks of cognitive training in working memory tasks (Tusch et al., 2016). Other studies related larger P300 amplitudes after cognitive training (O’Brien et al., 2013) and physical exercise (Kamijo et al., 2009) to increased attentional deployment and cognitive control, respectively.

The correlations between enhanced performance and increased P300 amplitude after anodal tDCS were conducted by including all participants that took part in the study (i.e., elderly and young). Thus, increased frontal activity after tDCS was related to improved performance also in young participants. The correlations between improved working memory and a larger frontal P300 amplitude in young participants were consistent with a previous study (Keeser et al., 2011) in which participants did exhibit a net improvement; however, the results of this abovementioned study should be interpreted with caution, as it involved a sample of 10 participants performing a 2-back task. In the present study, increased frontal P300 led to increased *d*′ in a subsample of young subjects whereas decreased frontal P300 led to decreased *d*′ in another subsample of young subjects, which explains the absence of a net improvement after anodal tDCS in the young group. In contrast, most of elderly participants exhibited increased P300 amplitude after anodal tDCS, which led to a net improvement after anodal tDCS in the elderly group. On the other hand, parietal P300 increased after all tDCS conditions between 350 and 450 ms, suggesting reduced difficulty in executing operations related to context information update after taking practice in the task (Polich, 2007). Moreover, the parietal P300 was larger in young than in elderly (350–450 ms) whereas the frontal P300 was larger in elderly than in young subjects (mainly in the anodal tDCS condition, see also the topographic maps, **Figures 3, 4**). These results are consistent with the reported P300 topographical changes related to aging (Friedman et al., 1997; Daffner et al., 2011; Saliasi et al., 2013; van Dinteren et al., 2014).

The frontal P300, whose increased amplitude correlated with improved performance after anodal tDCS, was related to the allocation of attentional resources to the upcoming stimulus, whereas the parietal P300 was related to context information update (Fabiani and Friedman, 1995; Friedman et al., 2001; Nieuwenhuis et al., 2005; Polich, 2007; Daffner et al., 2011; Wild-Wall et al., 2011; Saliasi et al., 2013; Tusch et al., 2016). Thus, these results indicate that increased working memory performance in elderly participants after anodal tDCS is related to enhanced attentional processes but not to improved efficiency in mental operations related to context information update. This finding aligns with previous studies that reported that encoding processes also depend on attentional capacity (Emrich and Ferber, 2012; Mazyar et al., 2012), and with studies that related the age-related decline in attentional capacity to greater susceptibility to interfering stimuli in working memory tasks (Schneider-Garces et al., 2010). Moreover, the correlations between improved working memory and enhanced bilateral frontal activity may be related to a previous behavioral study, which reported that left and right anodal tDCS equally improved working memory (Jones et al., 2015). These authors hypothesized that increased frontal activity mediates modulations of fronto-striatal connectivity, which leads to improved working memory. In line with this hypothesis, other studies reported increased striatal dopaminergic release after cognitive training (Backman et al., 2011; Kühn et al., 2011; Backman and Nyberg, 2013). Additionally, striatal modulations were related to transfer effects from cognitive training to untrained *n*-back tasks (Dahlin et al., 2008; Salminen et al., 2016).

The relationship between increased frontal activity and increased performance observed in the present study is consistent with the compensation-related utilization of neural circuits hypothesis (CRUNCH; Reuter-Lorenz and Cappell, 2008; Schneider-Garces et al., 2010; see also Cabeza et al., 2002; Davis et al., 2008; Daffner et al., 2011). This hypothesis predicts an inverted U-shaped relationship between task difficulty and allocation of neural resources such that neural resources increase at a higher task difficulty to maintain good performance. However, after achieving a critical point, which happens at lower difficulty levels in elderly than in young participants, additional increases in task difficulty are accompanied by a reduction in neural resources and impaired behavioral performance (Mattay et al., 2006; Wild-Wall et al., 2011). Considering that the tasks performed in the present study were highly demanding, it is possible that elderly participants were in the “descendent” slope of the inverted U-shaped curve hypothesized by the CRUNCH. Thus, the anodal tDCS favored “going backward” in the inverted-U curve hypothesized by this model, which would lead to increased brain activity and improved performance. Interestingly, other studies reporting heterogeneous results could fit within this model. For instance, Saliasi et al. (2013) reported correlations between higher frontal activation and worst performance in elderly subjects. Considering that a high allocation of neural activity to perform easy tasks was related to low brain resource levels (Reuter-Lorenz and Cappell, 2008; Schneider-Garces et al., 2010), the results of Saliasi et al. (2013) may be explained by the easy versions of the task that were used (i.e., 0-back and 1-back tasks). In contrast, studies reported reduced neural activity in highly demanding working memory tasks after cognitive training (Brehmer et al., 2011; Vermeij et al., 2017). In this case, the high number of cognitive training sessions implemented by these studies probably allowed a reduction in the subjective difficulty level even on highly demanding tasks.

A noteworthy limitation of the present study is the absence of an experimental condition to demonstrate that the observed effects are site specific, as suggested by recent reviews about non-invasive brain stimulation (Rossini et al., 2015). If anodal tDCS over a brain region not involved in the task (e.g., the vertex) failed to promote an increase in frontal activity, then we could have undoubtedly confirmed that increased frontal activity after anodal tDCS applied over the DLPFC is mediated by specific modulations of neural processes involved in task performance. However, if anodal tDCS over a brain region not involved in the task increases frontal activity, then we cannot exclude a non-specific increase in the arousal levels as the responsible mechanism for the observed frontal activity enhancement. Future studies should explore these alternative possibilities to further clarify the neural mechanisms underlying working memory improvement. Finally, another limitation of the present study is the small sample size, which might explain the weak tDCS effects that were observed on the behavioral data. Future studies should consider increasing the sample size. Increasing the sample size would be also useful to study the high inter-individual variability of the tDCS effects by dividing the samples in high and low performers, which is in line with recent studies about inter-individual variability of the tDCS effects (Tseng et al., 2012; Benwell et al., 2015; Hsu et al., 2016).

In summary, anodal tDCS applied over the left DLPFC increased the left frontal P300 amplitude in elderly participants. This increase was related to a tendency to improved working memory, as supported by a correlation analysis. Considering that frontal P300 amplitude is related to attentional processes, the results of the present study suggest that anodal tDCS can improve working memory by strengthening attentional processes. In contrast, anodal tDCS did not modulate the amplitude of the parietal P300, which is typically related to context update processes. In general, the present study suggests that anodal tDCS may have the capability to enhance working memory performance in healthy elderly subjects by promoting frontal compensatory mechanisms related to attentional processes.

## Author Contributions

JC designed and programmed the experimental task and procedures, collected and analyzed the data, interpreted the results, and wrote the manuscript. CR programmed the experimental task and procedures, collected and analyzed the data, and interpreted the results. PR interpreted the results and critically reviewed the manuscript. CM designed the experimental procedures and critically reviewed the manuscript. MP designed the experimental procedures, collected and analyzed the data, interpreted the results, and wrote the manuscript.

## Conflict of Interest Statement

The authors declare that the research was conducted in the absence of any commercial or financial relationships that could be construed as a potential conflict of interest.
